# Advanced Thinkers

**Published:** 1877-03

**Authors:** 


### "Advanced Thinkers."

That single expression—advanced thinkers—will do more to establish theories and doctrines, and practices also, than learning and experience and wisdom combined. Let a man who differs from the mass be pronounced by common consent an advanced thinker, his fame is established, no matter how extravagant his notions. To say that a man is an advanced thinker, means, as a general thing, that he don't think at all, but is captivated with some popular idea of some popular scientist, or that he aspires at notoriety by identifying himself with such. Time was when advanced thinking was a dangerous business, which men did not dare accept under penalty of martyrdom. Now it is the reverse. Advanced and original thinkers are really but few, and their advance is often more apparent than real. They often but exhume the old and instal it as new. Our age has much need of individual and independent thought. Men are afraid to hold fast to the past or the present in view of anything claiming to be in advance. It is in theology, in general science, in medicine. Better maxim than this was never uttered: "Prove all things—hold fast that which is good."—*Pacific Med. and Surg. Journal.*

